# Obinutuzumab combined with bendamustine for the treatment of hairy cell leukemia variant: a case report and literature review

**DOI:** 10.3389/fonc.2025.1635856

**Published:** 2025-08-18

**Authors:** Junjun Bai, Yi Zhang, Kangqi Lv, Bei Zhang, Hongxia Wang, Zhixin Pei, Yingxin Zhao, Jingjing Gu, Huimin Wu, Qinglin Song

**Affiliations:** ^1^ Department of Hematology, Jiaozuo People’s Hospital, Jiaozuo, Henan, China; ^2^ Department of Clinical Pharmacy, Jiaozuo People’s Hospital, Jiaozuo, Henan, China; ^3^ Laboratory of Hematological Diseases, Jiaozuo People’s Hospital, Jiaozuo, Henan, China

**Keywords:** hairy cell leukemia variant, obinutuzumab, bendamustine, targeted therapy, BRAF wild-type

## Abstract

Hairy cell leukemia variant (HCL-v) is a rare and more aggressive subtype of B-cell leukemia. While it shares certain clinical features with classical hairy cell leukemia (HCL-c), HCL-v typically follows a more malignant course and responds poorly to conventional therapies. We report a case of HCL-v in a 57-year-old male who was admitted with splenomegaly and abnormal blood counts. Based on bone marrow morphology and immunophenotyping, a diagnosis of HCL-v was established. The patient was treated with a combination of obinutuzumab and bendamustine. Following treatment, his blood counts normalized, spleen size significantly reduced, and bone marrow reassessment confirmed complete remission (CR) with minimal residual disease (MRD) negativity. The patient is currently undergoing maintenance therapy with obinutuzumab and remains in good clinical condition. This case demonstrates the promising clinical efficacy of obinutuzumab combined with bendamustine in treating HCL-v, suggesting its potential as a therapeutic option. However, long-term outcomes warrant further investigation.

## Introduction

Hairy cell leukemia variant (HCL-v) is a rare B-cell malignancy with distinct biological characteristics, accounting for approximately 10% of all hairy cell leukemia (HCL) cases. It predominantly affects elderly males and is clinically characterized by splenomegaly, peripheral lymphocytosis, and thrombocytopenia, typically without monocytopenia (1). Unlike classical HCL (HCL-c), tumor cells in HCL-v lack expression of CD25, CD123, and CD200, and do not harbor the BRAF V600E mutation (1–3). Moreover, patients with HCL-v often exhibit resistance to purine analogs such as cladribine and have a poorer prognosis. Although the current recommended treatment involves a combination of purine analogs and rituximab, the overall efficacy remains suboptimal (1). Therefore, the development of novel therapeutic strategies is crucial to improving outcomes in HCL-v.

In this report, we present a case of HCL-v successfully treated with obinutuzumab and bendamustine (the GB regimen), and review relevant literature to provide insights into potential new approaches for managing this challenging disease.

## Case presentation

In January 2024, a 57-year-old male was admitted to the hospital with a chief complaint of splenomegaly persisting for over six years. On admission, physical examination revealed stable vital signs, no signs of anemia, and no palpable superficial lymphadenopathy. The liver was not palpable below the costal margin, but the spleen was markedly enlarged, extending approximately 10 cm below the costal margin, non-tender to palpation. No edema was noted in the lower extremities. Complete blood count (CBC) showed leukocytosis with a white blood cell (WBC) count of 19.32 × 10^9^/L, red blood cell (RBC) count of 4.76 × 10^12^/L, hemoglobin (HGB) of 142 g/L, and a platelet (PLT) count of 76 × 10^9^/L, indicating hematologic abnormalities.

**Figure 1:**
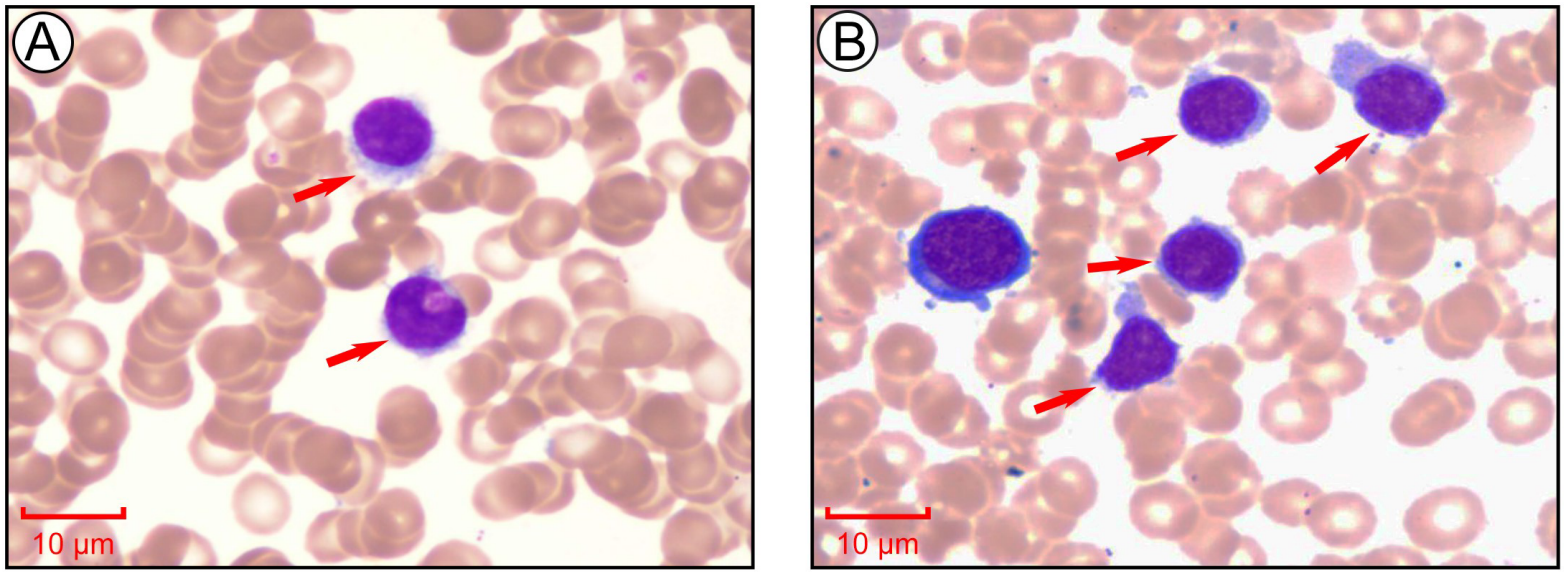
Morphological analysis of peripheral blood and bone marrow aspirate at the initial stage of diagnosis. **(A)** Morphological examination of peripheral blood cells via Giemsa staining. Images collected at 1000×, scale bar = 10 μm. **(B)** Morphological examination of bone marrow cells via Giemsa staining. Images collected at 1000×, scale bar = 10 μm.

**Figure 2:**
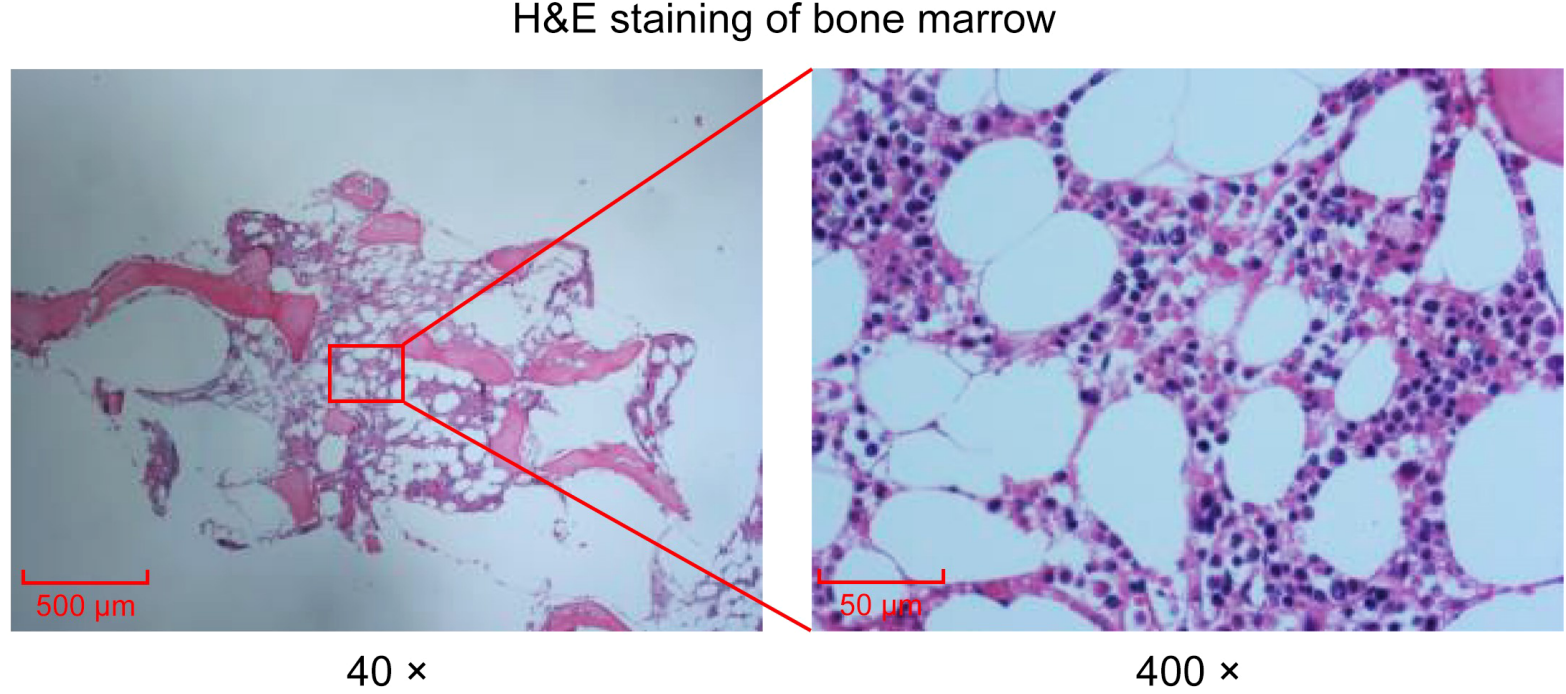
Histological examination of bone marrow via H&E staining. H&E staining of bone marrow. Images collected at 40× or 400×, scale bar = 500 μm or 50 μm.

**Figure 3:**
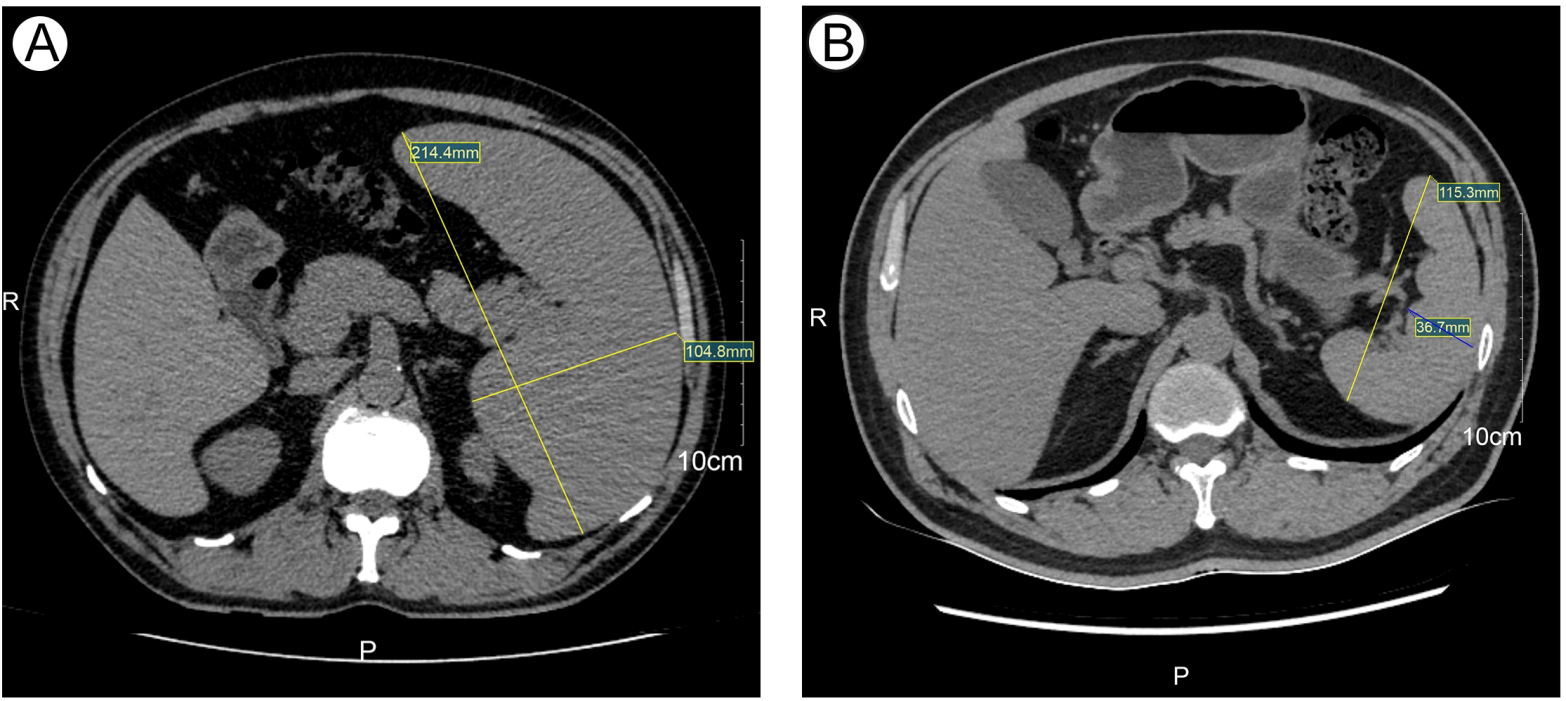
The size of Spleen at initial diagnosis and after treatment detected by CT. **(A)** Spleen size at initial diagnosis detected by CT. **(B)** Spleen size after treatment detected by CT.

Peripheral blood smear revealed marked leukocytosis with a decreased granulocyte ratio, morphologically normal granulocytes, and an increased lymphocyte percentage with the presence of atypical lymphocytes (**Figure 1A**). Bone marrow cytology showed markedly hypercellular marrow with a significantly increased lymphocyte proportion and abundant atypical lymphocytes. These cells were relatively small, with round nuclei, condensed chromatin, basophilic cytoplasm, occasional visible nucleoli, and cytoplasmic projections suggestive of “hairy” features (**Figure 1B**). Bone marrow biopsy revealed hypocellular marrow (approximately 30%) with increased abnormal lymphocytes (about 20%), distributed in clusters or scattered, occasionally within sinusoids. These cells were small to medium in size with scant to moderate cytoplasm, round to irregular nuclei, coarse chromatin, and decreased megakaryocytes, erythroid and granulocytic precursors were observed (**Figure 2**). Abdominal ultrasound and CT scans revealed severe splenomegaly, with dimensions of approximately 214.4 mm in length and 104.8 mm in thickness (**Figure 3A**).

**Figure 4:**
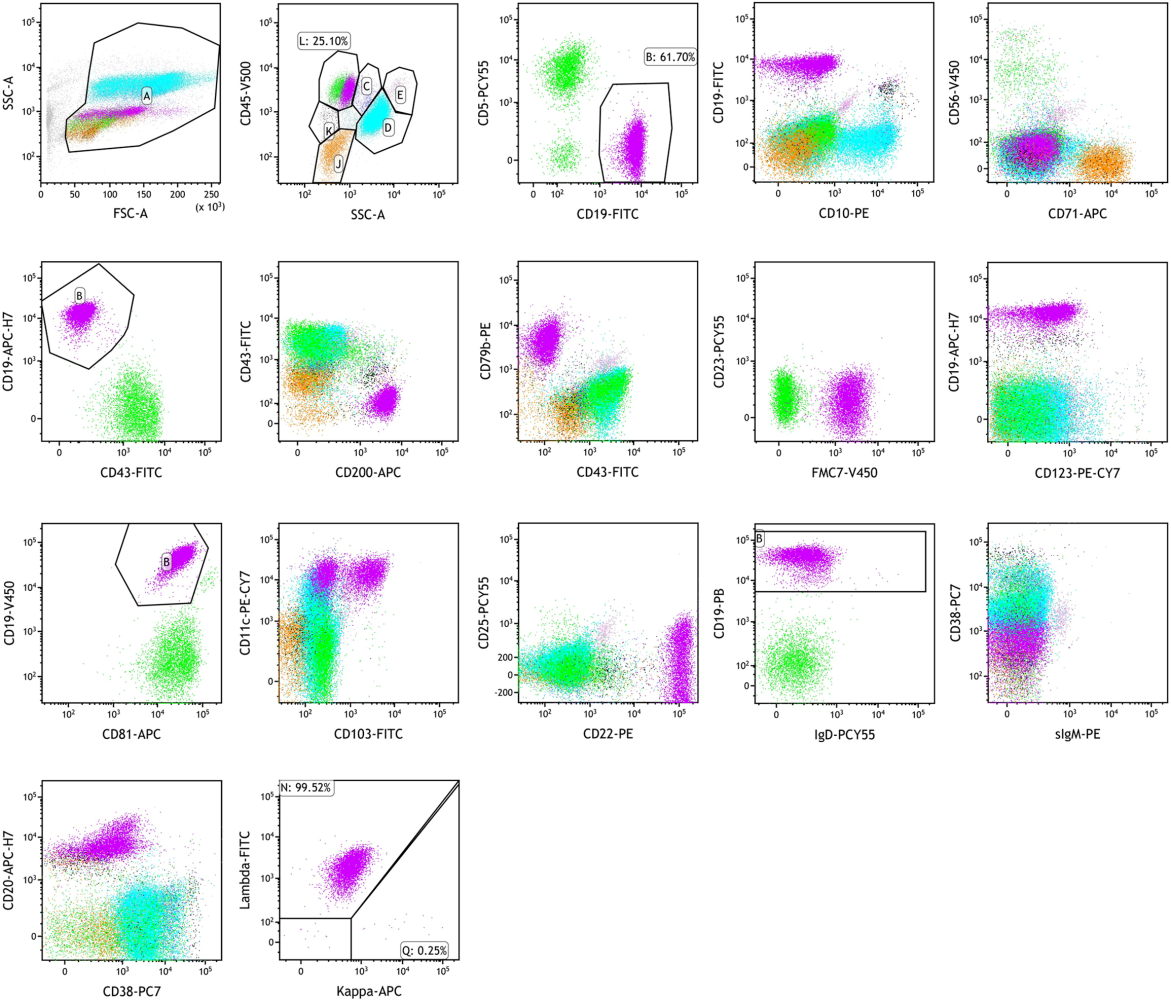
Flow cytometry analysis of a bone marrow aspirate. Bone marrow flow cytometry showing HCL-v diagnosis: strongly expressed CD19, FMC7, CD11c, and CD22; expressed CD200, CD79b, CD81, CD20, and surface lambda light chains; partially expressed CD103; negative for CD5, CD10, CD71, CD43, CD23, CD123, CD25, sIgD, sIgM, CD38, and kappa light chains.

Flow cytometric immunophenotyping of bone marrow cells revealed an abnormal leukemic B-cell population comprising 16.51% of nucleated cells. These cells strongly expressed CD19, FMC7, CD11c, and CD22; expressed CD200, CD79b, CD81, CD20, and surface lambda light chains; partially expressed CD103; and were negative for CD5, CD10, CD71, CD43, CD23, CD123, CD25, sIgD, sIgM, CD38, and kappa light chains (**Figure 4**). Further molecular and cytogenetic analysis showed a karyotype of 46, XY, del(14)(q24q32)[10]/46, XY[10]. BRAF V600E mutation testing was negative. However, IGH, IGK, and IGL gene rearrangements were positive, and the IGHV mutation frequency was 2.5%, classified as IGHV4–34 subtype. Next-generation sequencing (NGS) revealed a class I mutation in BCOR (p.H674Tf*66, 54.90%) and a class II mutation in ATM (p.L2519P, 8.50%). Based on the integration of morphologic, immunophenotypic, and molecular findings, the final diagnosis was hairy cell leukemia variant (HCL-v).

On January 20, 2024, the patient initiated chemotherapy with the GB regimen, consisting of obinutuzumab 1000 mg on days 1, 8, and 15, and bendamustine 175 mg on days 1-2. Following the first cycle, blood counts normalized. On February 19, 2024, a second cycle of the GB regimen was administered (obinutuzumab 1000 mg on day 0 and bendamustine 175 mg on days 1-2). Post-treatment evaluation showed complete remission (CR), minimal residual disease (MRD) negativity confirmed by flow cytometry (FCM) of bone marrow aspirates obtained after the second cycle, and normalization of spleen size (**Figure 3B**). The patient subsequently received four additional cycles of consolidation therapy with the GB regimen on March 16, April 15, May 14, and June 18, 2024. Follow-up assessments confirmed sustained CR. During chemotherapy, the patient experienced nausea (CTCAE grade 1). After the first and second cycles, mild leukopenia and thrombocytopenia (CTCAE grade 1) were observed. Subsequent treatments did not result in further declines in blood counts. The patient is currently receiving obinutuzumab maintenance therapy every three months for a planned duration of two years (total of eight doses).

## Discussion

Hairy cell leukemia-variant (HCL-v) is a rare and highly aggressive B-cell lymphoproliferative neoplasm that remains a significant clinical challenge. Unlike classical hairy cell leukemia (HCL-c), HCL-v typically lacks the BRAF V600E mutation, thereby limiting the use of BRAF inhibitors and complicating treatment strategies (4). While purine analog monotherapy or combination regimens with the anti-CD20 monoclonal antibody rituximab are standard treatments for HCL-c, HCL-v generally shows poor responses to these approaches. Therefore, developing novel therapeutic strategies is imperative. In the present case, the patient achieved complete remission (CR) and minimal residual disease (MRD) negativity following treatment with obinutuzumab in combination with bendamustine, suggesting that this regimen may represent a promising therapeutic option for HCL-v.

Clinically and biologically, HCL-v differs significantly from classical HCL. Immunophenotypically, HCL-v typically lacks hallmark markers such as CD25, CD123, and TRAP. Genetically, HCL-v is frequently associated with mutations in tumor suppressor genes such as TP53 and ATM, which may contribute to its resistance to standard therapies (4, 5). Previous studies have shown that combining purine analogs with rituximab results in higher response rates and longer progression-free survival (PFS) in HCL-v compared to monotherapy (5). Moreover, rituximab-based combinations with other agents, such as bendamustine, have demonstrated favorable efficacy in relapsed or refractory chronic lymphocytic leukemia and indolent B-cell lymphomas (6).

In this case, the patient tested negative for the BRAF V600E mutation, indicating that BRAF-targeted therapy was not appropriate. However, a p.L2519P mutation in the ATM gene was identified. ATM mutations are known to impair DNA repair pathways, which may lead to resistance to conventional chemotherapy (7). Bendamustine, a unique antineoplastic agent with both alkylating and purine analog properties, acts through DNA cross-linking and apoptosis induction. Notably, its cytotoxicity may be p53-independent, potentially overcoming resistance mechanisms associated with ATM mutations (8).

Obinutuzumab, a second-generation anti-CD20 monoclonal antibody engineered with glycoengineered Fc modifications to enhance antibody-dependent cellular cytotoxicity (ADCC), has demonstrated superior efficacy over rituximab in indolent lymphomas (9). In this case, the leukemia cells expressed high levels of CD20, making them an ideal target for obinutuzumab. The absence of CD25 and CD123 further suggested that conventional HCL-targeted strategies may not be effective in this patient, highlighting the potential advantage of obinutuzumab in such settings.

The combination of bendamustine and obinutuzumab has shown synergistic efficacy in indolent non-Hodgkin lymphoma and mantle cell lymphoma, particularly among patients refractory to rituximab, improving both response rates and duration of remission (10–12). However, data on the use of this combination in HCL-v remain limited. A large retrospective study analyzing 33 HCL-v cases reported an objective response rate (ORR) of 100% in patients receiving first-line therapy with purine nucleoside analogs plus rituximab (PNA+R). Notably, among those treated with bendamustine plus rituximab, three patients achieved MRD-negative CR (13). These findings support the rationale for exploring the efficacy of bendamustine combined with obinutuzumab in HCL-v. In this report, the patient achieved rapid hematologic recovery, normalization of splenic size, and MRD negativity after receiving the GB regimen (obinutuzumab + bendamustine), indicating a deep and durable response. This favorable outcome may result from the synergistic mechanisms of action: bendamustine induces DNA damage and enhances CD20 antigen expression, facilitating the activity of anti-CD20 antibodies; obinutuzumab, in turn, mediates effective clearance of residual leukemia cells via ADCC and complement-dependent cytotoxicity (CDC). This synergy may help overcome the treatment resistance often seen in HCL-v.

This deep and durable remission underscores the importance of sensitive disease monitoring and personalized therapeutic strategies in HCL-v. Advances in precision medicine have transformed the field of hematologic oncology, enabling many patients with blood cancers to receive individualized treatment based on molecular characteristics. In this context, minimal residual disease (MRD) monitoring plays a crucial role in assessing treatment response and guiding clinical decisions in both classical hairy cell leukemia (HCL) and its variant form (HCL-v). Currently, the most widely used methods for MRD assessment include bone marrow biopsy and flow cytometry. In this case, bone marrow MRD monitoring was performed using multiparameter flow cytometry (MFC), providing valuable information on treatment efficacy. Meanwhile, emerging techniques such as liquid biopsy based on circulating tumor DNA (ctDNA) are gaining increasing attention. This noninvasive approach offers the dual advantage of monitoring MRD while delivering genomic insights into residual leukemic clones. Recent reviews have underscored the utility of ctDNA-based MRD detection in B and T cell lymphomas, suggesting it may also hold promise for future application in HCL-v (14).

Molecular analysis revealed the presence of an IGHV4–34 immunoglobulin heavy-chain variable region gene mutation with a somatic hypermutation rate of 2.5%. IGHV4–34 is known for its unique biological characteristics across various B-cell malignancies and is frequently associated with autoimmune-driven B-cell clonal disorders (15, 16). Previous studies have linked IGHV4–34 with stereotyped B-cell receptor (BCR) usage in chronic lymphocytic leukemia, suggesting an antigen-driven clonal selection origin (10). IGHV4–34 has also been identified in mucosa-associated lymphoid tissue (MALT) lymphoma and diffuse large B-cell lymphoma (DLBCL), pointing toward a shared pathogenesis in IGHV4-34-positive B-cell tumors (15, 16). These findings underscore the biological and clinical relevance of IGHV4–34 across B-cell neoplasms.

Additionally, this patient harbored a BCOR p.H674Tf*66 mutation. BCOR, a gene involved in epigenetic regulation, is frequently inactivated in hematologic malignancies such as acute myeloid leukemia and myelodysplastic syndromes, and its mutations are often associated with poor prognosis (17, 18). The pathogenic mechanism likely involves dysfunction of the transcriptional repression complex, affecting signaling pathways that regulate cell proliferation and apoptosis (19–22). The presence of this mutation in our patient suggests a high-risk biological profile and further justifies the use of an intensified treatment strategy.

Despite the encouraging results, this case report has several limitations. First, the findings are based on a single patient, lacking validation from larger, multicenter studies. Second, longer follow-up is needed to assess the durability of response and progression-free survival. Third, the molecular profiling did not encompass the full spectrum of HCL-v-associated alterations, future work should expand genomic characterization to better inform precision therapy.

## Conclusion

In summary, we report a successful use of obinutuzumab combined with bendamustine in a patient with HCL-v who achieved CR and MRD negativity. This combination may offer an effective therapeutic strategy for BRAF wild-type, CD25-negative HCL-v patients. The mechanism likely involves bendamustine-induced DNA damage coupled with obinutuzumab-mediated immune clearance. Further multicenter clinical trials are warranted to validate the broad applicability of this regimen and to explore individualized treatment approaches guided by molecular subtyping.

## Data Availability

The datasets of this study are available from the corresponding author on reasonable request.

## References

[1] Matutes E Martínez-Trillos A Campo E . Hairy cell leukaemia-variant: Disease features and treatment. Best Pract Res Cl Ha. (2015) 28:253–63. doi: 10.1016/j.beha.2015.09.002, PMID: 26614904

[2] Pillai V Pozdnyakova O Charest K Li B Shahsafaei A Dorfman DM . CD200 flow cytometric assessment and semiquantitative immunohistochemical staining distinguishes hairy cell leukemia from hairy cell leukemia-variant and other B-cell lymphoproliferative disorders. Am J Clin Pathol. (2013) 140:536–43. doi: 10.1309/AJCPEBK31VQQNDDR, PMID: 24045551

[3] Salem DA Scott D McCoy CS Liewehr DJ Venzon DJ Arons E . Differential expression of CD43, CD81, and CD200 in classic versus variant hairy cell leukemia. Cytom Part B-Clin Cy. (2019) 96:275–82. doi: 10.1002/cyto.b.21785, PMID: 31077558 PMC8191384

[4] Paillassa J Safa F Troussard X . Updates in hairy cell leukemia (HCL) and variant-type HCL (HCL-V): rationale for targeted treatments with a focus on ibrutinib. Ther Adv Hematol. (2022) 13:1–14. doi: 10.1177/20406207221090886, PMID: 35450208 PMC9016521

[5] Wang T Wang Y Li R Yu Y Chen J Lyu R . Purine nucleoside analogs plus rituximab is an effective treatment choice for hairy cell leukemia-variant: results from a single center in China. Blood. (2021) 138:3547–7. doi: 10.1182/blood-2021-150141

[6] Weide R Feiten S Friesenhahn V Heymanns J Kleboth K Thomalla J . Retreatment with bendamustine-containing regimens in patients with relapsed/refractory chronic lymphocytic leukemia and indolent B-cell lymphomas achieves high response rates and some long lasting remissions. Leukemia Lymphoma. (2012) 54:1640–6. doi: 10.3109/10428194.2012.747679, PMID: 23151046

[7] Leoni LM Bailey B Reifert J Bendall HH Zeller RW Corbeil J . Bendamustine (Treanda) displays a distinct pattern of cytotoxicity and unique mechanistic features compared with other alkylating agents. Clin Cancer Res. (2008) 14:309–17. doi: 10.1158/1078-0432.CCR-07-1061, PMID: 18172283

[8] Garnock-Jones KP . Bendamustine: a review of its use in the management of indolent non-Hodgkin’s lymphoma and mantle cell lymphoma. Drugs. (2010) 70:1703–18. doi: 10.2165/11205860-000000000-00000, PMID: 20731477

[9] Herter S Herting F Mundigl O Waldhauer I Weinzierl T Fauti T . Preclinical activity of the type II CD20 antibody GA101 (obinutuzumab) compared with rituximab and ofatumumab *in vitro* and in xenograft models. Mol Cancer Ther. (2013) 12:2031–42. doi: 10.1158/1535-7163.MCT-12-1182, PMID: 23873847

[10] Kostareli E Hadzidimitriou A Stavroyianni N Darzentas N Athanasiadou A Gounari M . Molecular evidence for EBV and CMV persistence in a subset of patients with chronic lymphocytic leukemia expressing stereotyped IGHV4–34 B-cell receptors. Leukemia. (2009) 23:919–24. doi: 10.1038/leu.2008.379, PMID: 19148139

[11] Sehn LH Chua N Mayer J Dueck G Trněný M Bouabdallah K . Obinutuzumab plus bendamustine versus bendamustine monotherapy in patients with rituximab-refractory indolent non-Hodgkin lymphoma (GADOLIN): a randomised, controlled, open-label, multicentre, phase 3 trial. Lancet Oncol. (2016) 17:1081–93. doi: 10.1016/S1470-2045(16)30097-3, PMID: 27345636

[12] Robinson KS Williams ME van der Jagt RH Cohen P Herst JA Tulpule A . Phase II multicenter study of bendamustine plus rituximab in patients with relapsed indolent B-cell and mantle cell non-Hodgkin’s lymphoma. J Clin Oncol. (2008) 26:4473–9. doi: 10.1200/JCO.2008.17.0001, PMID: 18626004

[13] Wang Y Wang T Yu Y Wang Q Yan Y Li R . Purine nucleoside analogs plus rituximab are an effective treatment choice for hairy cell leukemia-variant. Ann Hematol. (2022) 101:1201–10. doi: 10.1007/s00277-022-04795-x, PMID: 35437610

[14] Almasri M Maher N Al Deeban B Diop NM Moia R Gaidano G . Liquid biopsy in B and T cell lymphomas: from bench to bedside. Int J Mol Sci. (2025) 26:4869. doi: 10.3390/ijms26104869, PMID: 40430009 PMC12112201

[15] Agathangelidis A Xochelli A Stamatopoulos K . A gene is known by the company it keeps: enrichment of TNFAIP3 gene aberrations in MALT lymphomas expressing IGHV4–34 antigen receptors. J Pathol. (2017) 243:403–6. doi: 10.1002/path.4982, PMID: 28892161

[16] Sebastián E Alcoceba M Balanzategui A Marín L Montes-Moreno S Flores T . Molecular characterization of immunoglobulin gene rearrangements in diffuse large B-cell lymphoma: antigen-driven origin and IGHV4–34 as a particular subgroup of the non-GCB subtype. Am J Pathol. (2012) 181:1879–88. doi: 10.1016/j.ajpath.2012.07.028, PMID: 22982190

[17] Cao Q Gearhart MD Gery S Shojaee S Yang H Sun H . BCOR regulates myeloid cell proliferation and differentiation. Leukemia. (2016) 30:1155–65. doi: 10.1038/leu.2016.2, PMID: 26847029 PMC5131645

[18] Damm F Chesnais V Nagata Y Yoshida K Scourzic L Okuno Y . BCOR and BCORL1 mutations in myelodysplastic syndromes and related disorders. Blood. (2013) 122:3169–77. doi: 10.1182/blood-2012-11-469619, PMID: 24047651

[19] Kommoss FK Chang KT Stichel D Banito A Jones DT Heilig CE . Endometrial stromal sarcomas with BCOR-rearrangement harbor MDM2 amplifications. J Pathol Clin Res. (2020) 6:178–84. doi: 10.1002/cjp2.165, PMID: 32352245 PMC7339170

[20] Bremer J Kottke R Johann PD von Hoff K Brazzola P Grotzer MA . A single supratentorial high-grade neuroepithelial tumor with two distinct BCOR mutations, exceptionally long complete remission and survival. Pediatr Blood Cancer. (2020) 67:e28384. doi: 10.1002/pbc.28384, PMID: 32383815

[21] Astolfi A Fiore M Melchionda F Indio V Bertuccio SN Pession A . BCOR involvement in cancer. Epigenomics-UK. (2019) 11:835–55. doi: 10.2217/epi-2018-0195, PMID: 31150281 PMC6595546

[22] Kang JH Lee SH Lee J Choi M Cho J Kim SJ . The mutation of BCOR is highly recurrent and oncogenic in mature T-cell lymphoma. BMC Cancer. (2021) 21:82. doi: 10.1186/s12885-021-07806-8, PMID: 33468080 PMC7816311

